# Retention in Care of Adult HIV Patients Initiating Antiretroviral Therapy in Tigray, Ethiopia: A Prospective Observational Cohort Study

**DOI:** 10.1371/journal.pone.0136117

**Published:** 2015-09-04

**Authors:** Raffaella Bucciardini, Vincenzo Fragola, Teshome Abegaz, Stefano Lucattini, Atakilt Halifom, Eskedar Tadesse, Micheal Berhe, Katherina Pugliese, Andrea Binelli, Paola De Castro, Roberta Terlizzi, Luca Fucili, Massimiliano Di Gregorio, Marco Mirra, Erika Olivieri, Tsigemariam Teklu, Teame Zegeye, Amanuel Haile, Stefano Vella, Loko Abraham, Hagos Godefay, CASA-project Health Facilities

**Affiliations:** 1 Istituto Superiore di Sanità, Rome, Italy; 2 College of Health Sciences, Mekelle University, Mekelle, Ethiopia; 3 Tigray Regional Health Bureau, Mekelle, Ethiopia; Infectious Disease Service, UNITED STATES

## Abstract

**Introduction:**

Although Ethiopia has been scaling up the antiretroviral therapy (ART) services, low retention in care of patients remains one of the main obstacles to treatment success. We report data on retention in care and its associated determinants in Tigray, Ethiopia.

**Methods:**

We used data from the CASA project, a prospective observational and multi-site study of a cohort of HIV-infected patients who initiated ART for the first time in Tigray. Four participating health facilities (HFs) located in the South of Tigray were considered for this study. Patients were followed for one year after ART initiation. The main outcome measure was represented by the current retention in care, defined as the proportion of patients who were alive and receiving ART at the same HF one year after ART initiation. Patients who started ART between January 1, 2013 and December 31, 2013 were included in this analysis. Patients were followed for one year after ART initiation. The determinants of retention were analysed using univariate and multivariate Cox Proportional Hazards model with robust sandwich estimates to account for within HF correlation.

**Results:**

The four participating HFs in Tigray were able to retain overall 85.1% of their patients after one year from starting ART. Loss to follow-up (5.5%) and transfers to other HF (6.6) were the main determinant of attrition. A multivariate analysis shows that the factors significantly associated with retention were the type of HF, gender and active TB. Alamata health center was the HF with the highest attrition rate (HR 2.99, 95% CI: 2.77–3.23). Active TB (HR 1.72, 95% CI: 1.23–2.41) and gender (HR 1.64, 95% CI: 1.10–2.56) were also significantly associated with attrition.

**Conclusions:**

Although Ethiopia has significantly improved access to the ART program, achieving and maintaining a satisfactory long-term retention rate is a future goal. This is difficult because of different retention rates among HFs. Moreover specific interventions should be directed to people of different sex to improve retention in care in male population.

## Introduction

HIV/AIDS is still one of the main health challenges to be faced in Ethiopia. Although HIV prevalence is not very high, and the country has recently experienced a major reduction in new cases of HIV infection, it still has a large number of people living with HIV (PLWH). In 2013 (the latest estimated data), there have been 793,700 PLHV including children, approximately 46,000 AIDS related deaths and about 900,000 AIDS orphans [1]. In 2011, according to the last Ethiopian Demographic Health Survey, the HIV adult prevalence was estimated at 1.5% (4.2% urban versus 0.6% rural; 1.9% women versus 1.0% men) [2].

In Tigray, the region where our study was conducted, the HIV adult prevalence is estimated at around 1.8%. Ethiopia has implemented, over the last decade, numerous valuable strategies to scale up antiretroviral therapy (ART) and improve the quality of HIV care. In 2003, the Government of Ethiopia introduced its ART programme with the goal of reducing HIV-related morbidity and mortality and, in 2005, started to provide free ART with the support of the U.S. President’s emergency Program for AIDS Relief (PEPFAR) and the

Global Fund to Fight Tuberculosis, AIDS and Malaria (GFTAM) [3–5]. Since 2006, the ART program has been decentralized to many health centers and hospitals [6].

According to the last estimates, at the end of 2013 the number of PLWH on treatment were 40% (317,443/ 793,700). Among HIV-infected adults 50% (298,512/593,400) were receiving ART while only 9,5% (18,931/200,300) of HIV-infected children were on ART [1].

In the third decade of the pandemic, despite the success of free ART services, Ethiopia is facing two major challenges: to increase ART coverage and the need for better management of patients on ART [7–8]. This latter issue relates primarily to patient retention in care, which is a key factor for adherence and retention on ART, and is associated with better immune reconstitution, increased survival and reduced HIV transmission [9–11]. This study aimed to investigate the retention in care and its associated determinants after one year of follow-up among patients who initiated on ART in Tigray, Ethiopia.

## Methods

### Study design, setting and participants

This study received ethics approval, and all patients provided written informed consent (*name of Ethic Committe*: *Mekelle University College of Health Sciences Research and Community Service Council*). The informed consent from patients 14–18 years of age was signed by adult relatives acting as guardians (immediate families like father or mother or next of kin) and not by patients themselves.

We used data from the CASA project, a prospective observational and multi-site ongoing study of a cohort of HIV-infected patients who initiated ART for the first time in Tigray. Four participating health facilities (HFs) were considered for this study, located in the South of Tigray, both in rural and urban areas. They include three health centers (Alamata, Mekelle and Mehoni health centers) and one referral hospital (Ayder hospital). The ART units of the three health centers are run by two or three nurses, one health officer and in some cases also by one infection disease specialist; while the referral hospital ART unit includes 2 medical directors and four nurses. Patients who started ART at these HFs between January 1, 2013 and December 31, 2013 were included in this analysis. Patients were followed for one year after ART initiation. Enrolled HIV-infected patients met the following inclusion criteria: 14 years of age or older; patients who started ART for the first time; patients who agreed to provide their home address and telephone contact.

### Data collection and outcome definition

After enrolment, participants were clinically followed according to the routine schedule of the HFs. Data were collected using forms (enrolment-form, follow-up-form, exit-form) specifically designed for this study. Forms were completed by the nurses working in the ART units of each HF, and included all data collected during the usual clinical practice. Data were entered, by trained CASA project personnel (case-managers), into a computerized database specifically created for the study. Finally, all data collected from each HF were aggregated and merged into a central multisite database.

Baseline demographic and clinical characteristics, including gender, age, religion, educational status, WHO clinical stage, haemoglobin level, body mass index (BMI, weight/height^2^: < = 18.5 = underweight, 18.6–25 = normal, >25 overweight), initial ART regimen, presence of tuberculosis (TB) coinfection and CD4+ cell counts at baseline, defined as the value available within six months from starting HIV treatment, were collected for all patients included in the cohort. Operational definitions of the outcomes were classified as follow:

- Loss to follow-up: patients who missed scheduled visit to the same HF more than three months after the last visit.
- Stop ART medication: patients known to have discontinued ART for any reasons.
- Mortality: patients recorded to be dead in the patient’s exit-form.
- Transfer out: patients formally transferred to another HF. Transferred patients were considered as retained in the initial HF until the date they were transferred out.
- Retain in care: patients who were alive and receiving ART at the same HF one year after ART initiation (does not include patients who were recorded as lost to follow-up, discontinued ART, deceased or transferred out) [12].
- Attrition for care: it is the opposite of retention. Patients who were lost to follow-up, had discontinued ART, were recorded as deceased or who had transferred out.

### Statistical analyses

Baseline characteristics were summarized by descriptive statistics. The main outcome measure was represented by retention in care in the study period, which encompasses the years 2013 and 2014. Kaplan-Meier method was used to estimate the probability of retention in care at different months of follow-up. Follow-up of patients lost to follow-up, stopped ART medication or transferred out was censored at the date of their last visit at the HF. Time to death was censored at the date of recorded death.

Univariate and multivariate Cox Proportional Hazards model with robust sandwich estimates to account for within HF correlation was used to identify factors associated with retention. Baseline variables utilized in the univariate model were: type of HF (Alamata health center, Mekelle health center, Mehoni health center, Ayder hospital), gender, age (< = 50 or >50), religion (orthodox or other religions), educational status (no education, primary, secondary, tertiary), BMI (< = 18.5, 18.6–25, > 25), WHO clinical stage (I/II or III/IV), CD4 cell count (<200 or > = 200), haemoglobin level (< = 10 or >10), active TB, and initial treatment regimen (efavirenz based or nevirapine based). Variables which resulted having significance in the univariate analysis were included in the multivariate model. We also performed a sensitivity analysis using a multiple imputation method to estimate the probability of being retained in care of patients who were transferred out to another ART unit. Analyses were performed using both the SPSS software, version 21.0 (SPSS Inc, Chicago, IL, USA) and the SAS statistical package, version 9.2 (SAS Institute, Inc., Cary, NC).

## Results

Overall, 563 patients were included in this study: 35.2%, 24.5%, 24.0% and 16.3% started on ART at Mehoni health center, Ayder hospital, Mekelle health center and Alamata health center, respectively. They were mostly females (66.4%) and of Orthodox religion (89.9). Median age was 32 years. Almost half of the people did not have any education status (43.9%). Most of the patients were clinically symptomatic (WHO III/IV: 53.5%). At start of ART, median CD4 cell count was 215 cells/μL. Almost all patients had a haemoglobin value of >10 g/dL (90.6%). A third of patients (34.3%) were underweight (BMI<18.5) and active TB was found in 6.4% of patients. The majority of patients received efavirenz-based regimens as first-line ART (79.8%) (Table 1).

**Table 1:** Baseline characteristics.

	Value at Baseline
**Health Facilities,** n **(%)**	
Mehoni health center	198 **(35.2)**
Ayder hospital	138 **(24.5)**
Mekelle health center	135 **(24.0)**
Alamata health center	92 **(16.3)**
**Sex,** n **(%)**	
Female	374 **(66.4)**
Male	189 **(33.6)**
**Age at start of ART (years),** mean±SD (n, range), median	**33**±9.5 (563,17–71),**32**
< = 50 n (%)	527 **(93.6)**
>50	36 **(6.4)**
**Religion,** n **(%)**	
Orthodox	506 **(89.9)**
Muslim	55 **(9.8)**
Protestant	2 **(0.4)**
**Educational status,** n **(%)**	
No education	247 (43.9)
Primary	190 (33.7)
Secondary	78 (13.9)
Tertiary	48 (8.5)
**BMI (kg/m** ^**2**^ **),** n **(%)**	
< = 18.5	193 **(34.3)**
18.6–25	342 **(60.7)**
>25	25 **(4.4)**
***Missing data*: *3***	
Clinical stage, n (%)	
WHO I-II	262 **(46.5)**
WHO III-IV	301 **(53.5)**
**CD4+ count (cells/μL),** mean ±SD (n, range), median	**232**±168 (551, 2–1724), **215**
<200 n (%)	240 **(42.6)**
> = 200	311 **(55.2)**
***Missing data*: *12***	
**Hemoglobin (g/dL),** n **(%)**	
< = 10	54 **(9.6)**
>10	509 **(90.4)**
**Hemoglobin (g/dL),** n **(%)**	
< = 10	54 **(9.6)**
>10	509 **(90.4)**
**Active TB,** n **(%)**	
Yes	36 **(6.4)**
No	527 **(93.6)**
**Initial treatment regimen,** n **(%)**	
Efavirenz based	449 **(79.8)**
Nevirapin based	114 **(20.2)**

**Fig 1:**
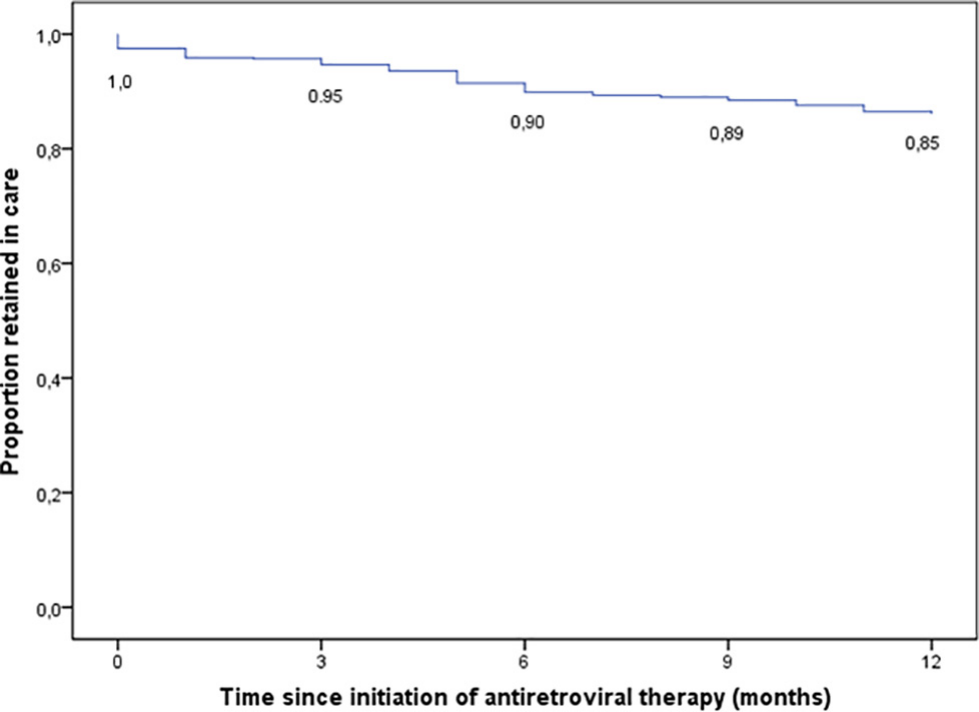
Kaplan-Meier estimate of retention in care at different months of follow-up on ART.

Probability of retention in care was 90.0% and 85.1% at 6 and 12 months (Fig 1). Total attrition rate was 14.9%: 5.5% of patients were lost to follow-up, 2.5% died, 0.4% stopped ART and 6.6% were transferred to another ART unit in Tigray (Table 2). A sensitivity analysis, which estimated the probability of being retained in care of patients transferred out, increased the retention in care from 85.1 to 90.9% (data not shown).

**Table 2:** Treatment outcomes of HIV+ patients one year after ART initiation, year 2013/2014

	All patients	Alamata health center	Ayder hospital	Mekelle health center	Mehoni health center
Started on ART	563	92	138	135	198
Alive and on ART at the same HF	479 (85.1)	72 (78.3)	127 (92.0)	111 (82.2)	169 (85.4)
Trasferred out to other HF^^^	37 (6.6)	4 (4.3)	7 (5.1)	11 (8.1)	15 (7.6)
Lost	31 (5.5)	12 (13.0)	1 (0.7)	8 (5.9)	10 (5.1)
Dead	14 (2.5)	3 (3.3)	3 (2.2)	4 (3.0)	4 (2.0)
Stopped ART	2 (0.4)	1 (1.1)	-	1 (0.7)	-
Attritioned for care	84 (14.9)	20 (21.7)	11 (8.0)	24 (17.8)	29 (14.6)

^^^ Transferred out were considered as retained in the initial HF until the date they were transferred out.

Finally, factors associated with attrition were investigated (Table 3). In the univariate analysis (taking Ayder hospital as the reference) we found that patients were less likely to be retained in care if they were cared by health centers. Alamata health center showed the highest attrition rate (HR 2.98, 95% CI: 2.93–3.04). Male gender was also associated with higher attrition (HR 1.61, 95% CI (1.17–2.21). Patients older than 50 years were more at risk of not returning to HF (HR 1.59, 95% CI: 1.11–2.29). Finally, patients with active TB showed a significant higher level of attrition (HR 2.15, 95% CI:1.95–2.37). Multivariate analysis was adjusted for type of HF, age, gender and active TB and confirmed Alamata health center as the HF with the highest attrition rate (HR 2.99, 95% CI: 2.77–3.23) and that active TB (HR 1.72, 95% CI: 1.23–2.41) and gender (HR 1.64, 95% CI: 1.10–2.56) were also significantly associated with attrition (Table 3).

**Table 3:** Cox proportional hazards model of association between baseline characteristics and retention in care

Baseline characteristics	Univariate analysis^Hazard ratio of attrition (95% CI)	Multivariate analysis^Hazard ratio of attrition (95% CI)
**Health Facility**		
Ayder hospital	Reference	Reference
Mehoni health center	1.95 (1.93–1.98)	2.27 (2.14–2.42)
Mekelle health center	2.36 (2.34–2.39)	2.58 (2.34–2.83)
Alamata health center	2.98 (2.93–3.04)	2.99 (2.77–3.23)
**Gender**		
Female	Reference	Reference
Male	1.61 (1.17–2.21)	1.64 (1.10–2.56)
**Age**		
< = 50	Reference	-
>50	1.59 (1.11–2.29)	
**Religion**		
Orthodox	Reference	-
Other religions	0.95 (0.46–1.97)	
**Educational status**		
No education	Reference	-
Primary	1.30 (0.92–1.85)	
Secondary	1.25 (0.57–2.78)	
Tertiary	0.75 (0.31–1.78)	
**BMI (kg/m** ^**2**^ **)**		
18.6–25 (normal)	Reference	-
< = 18.5 (underweight)	1.72 (0.89–3.31)	
>25 (overweight)	0.64 (0.29–1.39)	
**Clinical stage**		
WHO I-II	Reference	-
WHO III-IV	1.24 (0.75–2.03)	
**CD4 count (cells/μL)**		
< 200	Reference	-
> = 200	0.70 (0.56–0.86)	
**Hemoglobin (g/dL)**		
< = 10	Reference	-
>10	1.01 (0.56–1.84)	
**Active TB**		
No	Reference	Reference
Yes	2.15 (1.95–2.37)	1.72 (1.23–2.41)
**Initial treatment regimen**		
Efavirenz based	Reference	-
Nevirapin based	1.42 (0.89–2.27)	

^Hazard ratios estimated using robust sandwich estimators for variance to account for within HF correlation.

## Discussion

The results of this study show that the four participating HFs in Tigray were able to retain overall 85.1% of their patients after one year from starting ART. Loss to follow-up and transfers to other HF were the main determinants of attrition. The number of deaths was lower than estimates from national data and also lower than recorded mortality (7.4% 12 months after ART initiation) from a recently multi-clinic observational study carried out by Melaku et al in 56 HF in Ethiopia[1,13,14]. Our measure of mortality could be an underestimate of the true mortality, since some of the patients lost to follow-up, stopped ART or transferred out might have died. The rate of retention found in this study was slightly higher than that reported in other studies conducted in Ethiopia. Assefa et al, in a study conducted in 55 HFs in Ethiopia found that the retention rate at 12 months was 74% [15]. In another study carried out in North-West of Ethiopia the authors (Wubshet et al) showed that at the end of 12 months of follow-up, 73.8% of patients were retained in care [16]. It should be noted that the higher rate found in our study might be explained by the fact that the patients started ART in more recent years (2013/2014), when significant advances were introduced in the management of HIV patients, specifically regarding better first line ART regimens [7,17].

In fact, a more recent study of Assefa et al showed that retention rate increased from 77% in 2004/2005 to 92% in 2012/2013 [18]. This rather satisfactory retention rate can be explained by the fact that Ethiopia, and mainly Tigray, where the study was conducted, along with scaling up of ART has also implemented different strategies for improving retention in care, such as decentralization, task shifting and community-based organizations (CBO) patient’s support [9,18,19]. Decentralization of ART delivery has increased access to care, including those patients living in the more remote rural zones and has allowed patients to choose the structure closer to their residence [20,21]. The shortage of health personnel has been addressed by increasing task shifting from physician to nurses [20,22–23]. In addition, a large increase in CBOs initiatives have occurred during the last half-decade, to assist patients in dealing with social problems like stigma and in providing counselling and psychosocial support [21, 24–25].

Another relevant aspect in this study is the significant number of patients who were transferred out to another treatment site. This is common in Ethiopia, as well as in other sub-Saharan countries, as a consequence of the scaling-up of decentralization of ART delivery [12,15, 26–27].

Although we can speculate that transferred patients are still on ART at the facility to which they transferred to, it was not actually documented one year after ART initiation. For this reason we did not count in the primary analysis participant’s transferred to another clinic as retained as it could result in a overestimate of the patients retained in care at one year after ART initiations. An effective data linkage system between HFs can definitely help enhancing the reliability of outcomes.

In the multivariate analysis, we found that the factors significantly associated with attrition were type of HF, gender and the presence of active TB. Differences among HFs in rate of attrition were also found in two different studies [15, 21] conducted in Ethiopia, both showing that retention levels were considerably variable across HFs. The authors stated that HFs with higher retention rate had implemented a more comprehensive package of interventions aimed to improve retention in care, including activities performed by CBOs, a more intensive patient information on HIV disease and on the personal health benefit of being on ART. Since all HFs participating in this study have been implementing specific activities to improve patient retention in care, further studies will be carried out to better understand the reasons for the observed differences in retention. Moreover, some reports have shown that directly observed ART patients by CBOs was associated with improved adherence and retention [27–36]. For this reason, a greater involvement by CBOs should be encouraged, such as the implementation of a community-based HIV treatment program including community-based psychosocial support, HIV education, nutrition and transportation support, directly observed ART delivery and medical care by community health workers (CHWs).

Like other studies in sub-Saharan countries, being male was an independent factor associated with a higher attrition rate. Some reports have suggested that the reasons for this might be related to factors such as differences in health seeking behaviour, worse adherence to ART among men, national AIDS awareness initiatives focused more on women than on men [37–40].

Another factor associated with a higher rate of attrition was TB/HIV coinfection, probably due to the operational impact of associating TB treatment to ART, despite the existing good linkage between TB and HIV care in Tigray. Indeed, TB remains one of the most important public health problems in Ethiopia, exacerbated by the presence of a significant number of PLWH. A full integration of the services for the concomitant treatment of HIV-related co-infections is still a major challenge [41–42].

This study has limitations. The number of HFs and patients included in this analysis was small and the observed time of follow-up was short. Although Ethiopia has significantly improved access to the ART program, achieving and maintaining a satisfactory retention rate in long term remain a key challenge. As documented in some studies, carried out in Ethiopia as well as in other sub-Saharan countries, retention rates decrease over time. In the aforementioned article, conducted on 55 HFs in Ethiopia, Assefa et al showed that after 6, 12, and 24 months on ART retention rate was 80%, 74% and 68% respectively. Similar results were published in other studies, like a systematic review in sub-Saharan Africa by Fox and Rosen [10], showing retention in care declining from 80.2% at 12 months to 76.8% at 24 months and 72.3% at 36 months. Moreover, despite the involvement of the local CBOs in tracing the patients who missed their scheduled follow-up visit, the information on how many patients were traced and the reasons for not returning to visit were not strictly reported. For this reason patients lost of follow-up may also have died or have stopped taking ART.

The competitive advantage of the present study is that, differently from published reports where data collection has been prevalently retrospective, our retention rate calculations are based on a longitudinal observational study of a prospective cohort which is ongoing and will be followed for at least 5 years.

This article only provides preliminary results on a short period of time and with few participating HFs. Additional analyses will be implemented in the next future.

## Supporting Information

S1 Data Dataset for Table 1. (SAV)

S2 Data Dataset for Table 2. (SAV)

S3 Data Dataset for Table 3 and Fig 1. (SAV)
