# Correction: Retention in Care of Adult HIV Patients Initiating Antiretroviral Therapy in Tigray, Ethiopia: A Prospective Observational Cohort Study

**DOI:** 10.1371/journal.pone.0139428

**Published:** 2015-09-25

**Authors:** The PLOS ONE Staff

## Notice of Republication

This article was republished on September 15, 2015, to replace the name of the 21st author, Hagos Godefay, which was missing from the byline. The publisher apologizes for the error. Please download this article again to view the correct version. The originally published, uncorrected article and the republished, corrected article are provided here for reference.

## Supporting Information

S1 File Originally published, uncorrected article. (PDF)

S2 File Republished, corrected article. (PDF)
