# Supplementary material for: Correction: Retention in Care of Adult HIV Patients Initiating Antiretroviral Therapy in Tigray, Ethiopia: A Prospective Observational Cohort Study
Source: PLoS One. 2015 Sep 25;10(9):e0139428. doi: 10.1371/journal.pone.0139428 (PMC4583283; doi:10.1371/journal.pone.0139428)
Supplement: S2 File — (PDF) [file pone.0139428.s002.pdf]

PLOS ONE

RESEARCH ARTICLE

Retention in Care of Adult HIV Patients Initiating Antiretroviral Therapy in Tigray, Ethiopia: A Prospective Observational Cohort Study

Raffaella Bucciardini[1]*, Vincenzo Fragola[1], Teshome Abegaz[2], Stefano Lucattini[1], Atakilt Halifom[3], Eskedar Tadesse[2], Micheal Berhe[2], Katherina Pugliese[1], Andrea Binelli[1], Paola De Castro[1], Roberta Terlizzi[1], Luca Fucili[1], Massimiliano Di Gregorio[1], Marco Mirra[1], Erika Olivieri[1], Tsigemariam Teklu[3], Teame Zegeye[3], Amanuel Haile[2], Stefano Vella[1], Loko Abraham[2], Hagos Godefay[3], CASA-project Health Facilities[3]

1 Istituto Superiore di Sanità, Rome, Italy, **2** College of Health Sciences, Mekelle University, Mekelle, Ethiopia, **3** Tigray Regional Health Bureau, Mekelle, Ethiopia

* raffaella.bucciardini@iss.it

CrossMark
click for updates

OPEN ACCESS

Citation: Bucciardini R, Fragola V, Abegaz T, Lucattini S, Halifom A, Tadesse E, et al. (2015) Retention in Care of Adult HIV Patients Initiating Antiretroviral Therapy in Tigray, Ethiopia: A Prospective Observational Cohort Study. PLoS ONE 10(9): e0136117. doi:10.1371/journal.pone.0136117

Editor: Jason F Okulicz, Infectious Disease Service, UNITED STATES

Received: May 26, 2015

Accepted: July 29, 2015

Published: September 4, 2015

Copyright: © 2015 Bucciardini et al. This is an open access article distributed under the terms of the Creative Commons Attribution License, which permits unrestricted use, distribution, and reproduction in any medium, provided the original author and source are credited.

Data Availability Statement: Due to containing identifying patient information, data are available upon request from Raffaella Bucciardini: raffaella.bucciardini@iss.it.

Funding: This work was supported by the Italian Ministry of Health—Department of Prevention and Innovation, Rome, Italy. The funder had no role in study design, data collection and analysis, decision to publish, or preparation of the manuscript.

Competing Interests: The authors have declared that no competing interests exist.

Abstract

Introduction

Although Ethiopia has been scaling up the antiretroviral therapy (ART) services, low retention in care of patients remains one of the main obstacles to treatment success. We report data on retention in care and its associated determinants in Tigray, Ethiopia.

Methods

We used data from the CASA project, a prospective observational and multi-site study of a cohort of HIV-infected patients who initiated ART for the first time in Tigray. Four participating health facilities (HFs) located in the South of Tigray were considered for this study. Patients were followed for one year after ART initiation. The main outcome measure was represented by the current retention in care, defined as the proportion of patients who were alive and receiving ART at the same HF one year after ART initiation. Patients who started ART between January 1, 2013 and December 31, 2013 were included in this analysis. Patients were followed for one year after ART initiation. The determinants of retention were analysed using univariate and multivariate Cox Proportional Hazards model with robust sandwich estimates to account for within HF correlation.

Results

The four participating HFs in Tigray were able to retain overall 85.1% of their patients after one year from starting ART. Loss to follow-up (5.5%) and transfers to other HF (6.6) were the main determinant of attrition. A multivariate analysis shows that the factors significantly associated with retention were the type of HF, gender and active TB. Alamata health center was the HF with the highest attrition rate (HR 2.99, 95% CI: 2.77–3.23). Active TB (HR 1.72,

95% CI: 1.23–2.41) and gender (HR 1.64, 95% CI: 1.10–2.56) were also significantly associated with attrition.

Conclusions

Although Ethiopia has significantly improved access to the ART program, achieving and maintaining a satisfactory long-term retention rate is a future goal. This is difficult because of different retention rates among HFs. Moreover specific interventions should be directed to people of different sex to improve retention in care in male population.

Introduction

HIV/AIDS is still one of the main health challenges to be faced in Ethiopia. Although HIV prevalence is not very high, and the country has recently experienced a major reduction in new cases of HIV infection, it still has a large number of people living with HIV (PLWH). In 2013 (the latest estimated data), there have been 793,700 PLHV including children, approximately 46,000 AIDS related deaths and about 900,000 AIDS orphans [1]. In 2011, according to the last Ethiopian Demographic Health Survey, the HIV adult prevalence was estimated at 1.5% (4.2% urban versus 0.6% rural; 1.9% women versus 1.0% men) [2].

In Tigray, the region where our study was conducted, the HIV adult prevalence is estimated at around 1.8%. Ethiopia has implemented, over the last decade, numerous valuable strategies to scale up antiretroviral therapy (ART) and improve the quality of HIV care. In 2003, the Government of Ethiopia introduced its ART programme with the goal of reducing HIV-related morbidity and mortality and, in 2005, started to provide free ART with the support of the U.S. President's emergency Program for AIDS Relief (PEPFAR) and the

Global Fund to Fight Tuberculosis, AIDS and Malaria (GFTAM) [3–5]. Since 2006, the ART program has been decentralized to many health centers and hospitals [6].

According to the last estimates, at the end of 2013 the number of PLWH on treatment were 40% (317,443/ 793,700). Among HIV-infected adults 50% (298,512/593,400) were receiving ART while only 9,5% (18,931/200,300) of HIV-infected children were on ART [1].

In the third decade of the pandemic, despite the success of free ART services, Ethiopia is facing two major challenges: to increase ART coverage and the need for better management of patients on ART [7–8]. This latter issue relates primarily to patient retention in care, which is a key factor for adherence and retention on ART, and is associated with better immune reconstitution, increased survival and reduced HIV transmission [9–11]. This study aimed to investigate the retention in care and its associated determinants after one year of follow-up among patients who initiated on ART in Tigray, Ethiopia.

Methods

Study design, setting and participants

This study received ethics approval, and all patients provided written informed consent (*name of Ethic Committe*: *Mekelle University College of Health Sciences Research and Community Service Council*). The informed consent from patients 14–18 years of age was signed by adult relatives acting as guardians (immediate families like father or mother or next of kin) and not by patients themselves.

We used data from the CASA project, a prospective observational and multi-site ongoing study of a cohort of HIV-infected patients who initiated ART for the first time in Tigray. Four participating health facilities (HFs) were considered for this study, located in the South of Tigray, both in rural and urban areas. They include three health centers (Alamata, Mekelle and Mehoni health centers) and one referral hospital (Ayder hospital). The ART units of the three health centers are run by two or three nurses, one health officer and in some cases also by one infection disease specialist; while the referral hospital ART unit includes 2 medical directors and four nurses. Patients who started ART at these HFs between January 1, 2013 and December 31, 2013 were included in this analysis. Patients were followed for one year after ART initiation. Enrolled HIV-infected patients met the following inclusion criteria: 14 years of age or older; patients who started ART for the first time; patients who agreed to provide their home address and telephone contact.

Data collection and outcome definition

After enrolment, participants were clinically followed according to the routine schedule of the HFs. Data were collected using forms (enrolment-form, follow-up-form, exit-form) specifically designed for this study. Forms were completed by the nurses working in the ART units of each HF, and included all data collected during the usual clinical practice. Data were entered, by trained CASA project personnel (case-managers), into a computerized database specifically created for the study. Finally, all data collected from each HF were aggregated and merged into a central multisite database.

Baseline demographic and clinical characteristics, including gender, age, religion, educational status, WHO clinical stage, haemoglobin level, body mass index (BMI, weight/height^2: $<= 18.5$ = underweight, 18.6–25 = normal, >25 overweight), initial ART regimen, presence of tuberculosis (TB) coinfection and CD4+ cell counts at baseline, defined as the value available within six months from starting HIV treatment, were collected for all patients included in the cohort. Operational definitions of the outcomes were classified as follow:

- Loss to follow-up: patients who missed scheduled visit to the same HF more than three months after the last visit.
- Stop ART medication: patients known to have discontinued ART for any reasons.
- Mortality: patients recorded to be dead in the patient's exit-form.
- Transfer out: patients formally transferred to another HF. Transferred patients were considered as retained in the initial HF until the date they were transferred out.
- Retain in care: patients who were alive and receiving ART at the same HF one year after ART initiation (does not include patients who were recorded as lost to follow-up, discontinued ART, deceased or transferred out) [12].
- Attrition for care: it is the opposite of retention. Patients who were lost to follow-up, had discontinued ART, were recorded as deceased or who had transferred out.

Statistical analyses

Baseline characteristics were summarized by descriptive statistics. The main outcome measure was represented by retention in care in the study period, which encompasses the years 2013 and 2014. Kaplan-Meier method was used to estimate the probability of retention in care at different months of follow-up. Follow-up of patients lost to follow-up, stopped ART medication

or transferred out was censored at the date of their last visit at the HF. Time to death was censored at the date of recorded death.

Univariate and multivariate Cox Proportional Hazards model with robust sandwich estimates to account for within HF correlation was used to identify factors associated with retention. Baseline variables utilized in the univariate model were: type of HF (Alamata health center, Mekelle health center, Mehoni health center, Ayder hospital), gender, age (< = 50 or >50), religion (orthodox or other religions), educational status (no education, primary, secondary, tertiary), BMI (< = 18.5, 18.6–25, > 25), WHO clinical stage (I/II or III/IV), CD4 cell count (<200 or > = 200), haemoglobin level (< = 10 or >10), active TB, and initial treatment regimen (efavirenz based or nevirapine based). Variables which resulted having significance in the univariate analysis were included in the multivariate model. We also performed a sensitivity analysis using a multiple imputation method to estimate the probability of being retained in care of patients who were transferred out to another ART unit. Analyses were performed using both the SPSS software, version 21.0 (SPSS Inc, Chicago, IL, USA) and the SAS statistical package, version 9.2 (SAS Institute, Inc., Cary, NC).

Results

Overall, 563 patients were included in this study: 35.2%, 24.5%, 24.0% and 16.3% started on ART at Mehoni health center, Ayder hospital, Mekelle health center and Alamata health center, respectively. They were mostly females (66.4%) and of Orthodox religion (89.9). Median age was 32 years. Almost half of the people did not have any education status (43.9%). Most of the patients were clinically symptomatic (WHO III/IV: 53.5%). At start of ART, median CD4 cell count was 215 cells/μL. Almost all patients had a haemoglobin value of >10 g/dL (90.6%). A third of patients (34.3%) were underweight (BMI<18.5) and active TB was found in 6.4% of patients. The majority of patients received efavirenz-based regimens as first-line ART (79.8%) (Table 1).

Probability of retention in care was 90.0% and 85.1% at 6 and 12 months (Fig 1). Total attrition rate was 14.9%: 5.5% of patients were lost to follow-up, 2.5% died, 0.4% stopped ART and 6.6% were transferred to another ART unit in Tigray (Table 2). A sensitivity analysis, which estimated the probability of being retained in care of patients transferred out, increased the retention in care from 85.1 to 90.9% (data not shown).

Finally, factors associated with attrition were investigated (Table 3). In the univariate analysis (taking Ayder hospital as the reference) we found that patients were less likely to be retained in care if they were cared by health centers. Alamata health center showed the highest attrition rate (HR 2.98, 95% CI: 2.93–3.04). Male gender was also associated with higher attrition (HR 1.61, 95% CI (1.17–2.21). Patients older than 50 years were more at risk of not returning to HF (HR 1.59, 95% CI: 1.11–2.29). Finally, patients with active TB showed a significant higher level of attrition (HR 2.15, 95% CI:1.95–2.37). Multivariate analysis was adjusted for type of HF, age, gender and active TB and confirmed Alamata health center as the HF with the highest attrition rate (HR 2.99, 95% CI: 2.77–3.23) and that active TB (HR 1.72, 95% CI: 1.23–2.41) and gender (HR 1.64, 95% CI: 1.10–2.56) were also significantly associated with attrition (Table 3).

Discussion

The results of this study show that the four participating HFs in Tigray were able to retain overall 85.1% of their patients after one year from starting ART. Loss to follow-up and transfers to other HF were the main determinants of attrition. The number of deaths was lower than estimates from national data and also lower than recorded mortality (7.4% 12 months after ART initiation) from a recently multi-clinic observational study carried out by Melaku et al in 56 HF

Table 1. Baseline characteristics.

	Value at Baseline
**Health Facilities,** n **(%)**	
Mehoni health center	198 **(35.2)**
Ayder hospital	138 **(24.5)**
Mekelle health center	135 **(24.0)**
Alamata health center	92 **(16.3)**
**Sex,** n **(%)**	
Female	374 **(66.4)**
Male	189 **(33.6)**
**Age at start of ART (years),** mean±SD (n, range), median	**33**±9.5 (563,17–71),**32**
< = 50 n (%)	527 **(93.6)**
>50	36 **(6.4)**
**Religion,** n **(%)**	
Orthodox	506 **(89.9)**
Muslim	55 **(9.8)**
Protestant	2 **(0.4)**
**Educational status,** n **(%)**	
No education	247 (43.9)
Primary	190 (33.7)
Secondary	78 (13.9)
Tertiary	48 (8.5)
**BMI (kg/m^2),** n **(%)**	
< = 18.5	193 **(34.3)**
18.6–25	342 **(60.7)**
>25	25 **(4.4)**
Missing data*: *3	
Clinical stage, n (%)	
WHO I-II	262 **(46.5)**
WHO III-IV	301 **(53.5)**
**CD4+ count (cells/μL),** mean ±SD (n, range), median	**232**±168 (551, 2–1724), **215**
<200 n (%)	240 **(42.6)**
> = 200	311 **(55.2)**
Missing data*: *12	
**Hemoglobin (g/dL),** n **(%)**	
< = 10	54 **(9.6)**
>10	509 **(90.4)**
**Hemoglobin (g/dL),** n **(%)**	
< = 10	54 **(9.6)**
>10	509 **(90.4)**
**Active TB,** n **(%)**	
Yes	36 **(6.4)**
No	527 **(93.6)**
**Initial treatment regimen,** n **(%)**	
Efavirenz based	449 **(79.8)**
Nevirapin based	114 **(20.2)**

doi:10.1371/journal.pone.0136117.t001

in Ethiopia[1,13,14]. Our measure of mortality could be an underestimate of the true mortality, since some of the patients lost to follow-up, stopped ART or transferred out might have died.

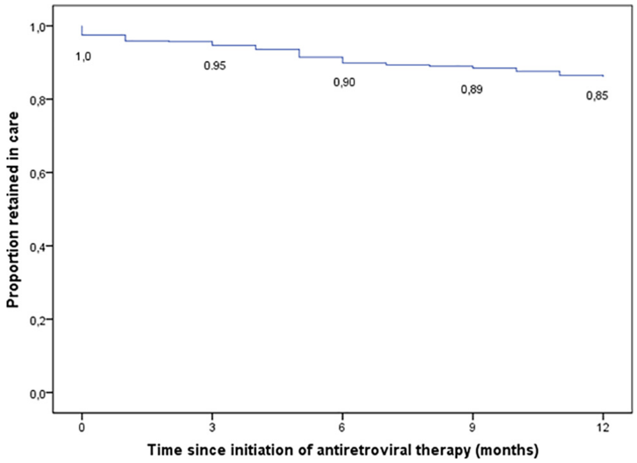

Fig 1. Kaplan-Meier estimate of retention in care at different months of follow-up on ART.

doi:10.1371/journal.pone.0136117.g001

The rate of retention found in this study was slightly higher than that reported in other studies conducted in Ethiopia. Assefa et al, in a study conducted in 55 HFs in Ethiopia found that the retention rate at 12 months was 74% [15]. In another study carried out in North-West of Ethiopia the authors (Wubshet et al) showed that at the end of 12 months of follow-up, 73.8% of patients were retained in care [16]. It should be noted that the higher rate found in our study might be explained by the fact that the patients started ART in more recent years (2013/2014), when significant advances were introduced in the management of HIV patients, specifically regarding better first line ART regimens [7,17].

In fact, a more recent study of Assefa et al showed that retention rate increased from 77% in 2004/2005 to 92% in 2012/2013 [18]. This rather satisfactory retention rate can be explained by the fact that Ethiopia, and mainly Tigray, where the study was conducted, along with scaling up of ART has also implemented different strategies for improving retention in care, such as decentralization, task shifting and community-based organizations (CBO) patient's support [9,18,19]. Decentralization of ART delivery has increased access to care, including those patients living in the more remote rural zones and has allowed patients to choose the structure closer to their residence [20,21]. The shortage of health personnel has been addressed by increasing task shifting from physician to nurses [20,22–23]. In addition, a large increase in CBOs initiatives have occurred during the last half-decade, to assist patients in dealing with social problems like stigma and in providing counselling and psychosocial support [21, 24–25].

Table 2. Treatment outcomes of HIV+ patients one year after ART initiation, year 2013/2014

	All patients	Alamata health center	Ayder hospital	Mekelle health center	Mehoni health center
Started on ART	563	92	138	135	198
Alive and on ART at the same HF	479 (85.1)	72 (78.3)	127 (92.0)	111 (82.2)	169 (85.4)
Trasferred out to other HF^	37 (6.6)	4 (4.3)	7 (5.1)	11 (8.1)	15 (7.6)
Lost	31 (5.5)	12 (13.0)	1 (0.7)	8 (5.9)	10 (5.1)
Dead	14 (2.5)	3 (3.3)	3 (2.2)	4 (3.0)	4 (2.0)
Stopped ART	2 (0.4)	1 (1.1)	-	1 (0.7)	-
Attritioned for care	84 (14.9)	20 (21.7)	11 (8.0)	24 (17.8)	29 (14.6)

^ Transferred out were considered as retained in the initial HF until the date they were transferred out.

doi:10.1371/journal.pone.0136117.t002

Table 3. Cox proportional hazards model of association between baseline characteristics and retention in care

Baseline characteristics	Univariate analysis^Hazard ratio of attrition (95% CI)	Multivariate analysis^Hazard ratio of attrition (95% CI)
**Health Facility**		
Ayder hospital	Reference	Reference
Mehoni health center	1.95 (1.93–1.98)	2.27 (2.14–2.42)
Mekelle health center	2.36 (2.34–2.39)	2.58 (2.34–2.83)
Alamata health center	2.98 (2.93–3.04)	2.99 (2.77–3.23)
**Gender**		
Female	Reference	Reference
Male	1.61 (1.17–2.21)	1.64 (1.10–2.56)
**Age**		
< = 50	Reference	-
>50	1.59 (1.11–2.29)	
**Religion**		
Orthodox	Reference	-
Other religions	0.95 (0.46–1.97)	
**Educational status**		
No education	Reference	-
Primary	1.30 (0.92–1.85)	
Secondary	1.25 (0.57–2.78)	
Tertiary	0.75 (0.31–1.78)	
**BMI (kg/m^2)**		
18.6–25 (normal)	Reference	-
< = 18.5 (underweight)	1.72 (0.89–3.31)	
>25 (overweight)	0.64 (0.29–1.39)	
**Clinical stage**		
WHO I-II	Reference	-
WHO III-IV	1.24 (0.75–2.03)	
**CD4 count (cells/μL)**		
< 200	Reference	-
> = 200	0.70 (0.56–0.86)	
**Hemoglobin (g/dL)**		
< = 10	Reference	-
>10	1.01 (0.56–1.84)	
**Active TB**		
No	Reference	Reference
Yes	2.15 (1.95–2.37)	1.72 (1.23–2.41)
**Initial treatment regimen**		
Efavirenz based	Reference	-
Nevirapin based	1.42 (0.89–2.27)	

^Hazard ratios estimated using robust sandwich estimators for variance to account for within HF correlation.

doi:10.1371/journal.pone.0136117.t003

Another relevant aspect in this study is the significant number of patients who were transferred out to another treatment site. This is common in Ethiopia, as well as in other sub-Saharan countries, as a consequence of the scaling-up of decentralization of ART delivery [12,15,26–27].

Although we can speculate that transferred patients are still on ART at the facility to which they transferred to, it was not actually documented one year after ART initiation. For this

reason we did not count in the primary analysis participant's transferred to another clinic as retained as it could result in a overestimate of the patients retained in care at one year after ART initiations. An effective data linkage system between HFs can definitely help enhancing the reliability of outcomes.

In the multivariate analysis, we found that the factors significantly associated with attrition were type of HF, gender and the presence of active TB. Differences among HFs in rate of attrition were also found in two different studies [15, 21] conducted in Ethiopia, both showing that retention levels were considerably variable across HFs. The authors stated that HFs with higher retention rate had implemented a more comprehensive package of interventions aimed to improve retention in care, including activities performed by CBOs, a more intensive patient information on HIV disease and on the personal health benefit of being on ART. Since all HFs participating in this study have been implementing specific activities to improve patient retention in care, further studies will be carried out to better understand the reasons for the observed differences in retention. Moreover, some reports have shown that directly observed ART patients by CBOs was associated with improved adherence and retention [27–36]. For this reason, a greater involvement by CBOs should be encouraged, such as the implementation of a community-based HIV treatment program including community-based psychosocial support, HIV education, nutrition and transportation support, directly observed ART delivery and medical care by community health workers (CHWs).

Like other studies in sub-Saharan countries, being male was an independent factor associated with a higher attrition rate. Some reports have suggested that the reasons for this might be related to factors such as differences in health seeking behaviour, worse adherence to ART among men, national AIDS awareness initiatives focused more on women than on men [37–40].

Another factor associated with a higher rate of attrition was TB/HIV coinfection, probably due to the operational impact of associating TB treatment to ART, despite the existing good linkage between TB and HIV care in Tigray. Indeed, TB remains one of the most important public health problems in Ethiopia, exacerbated by the presence of a significant number of PLWH. A full integration of the services for the concomitant treatment of HIV-related co-infections is still a major challenge [41–42].

This study has limitations. The number of HFs and patients included in this analysis was small and the observed time of follow-up was short. Although Ethiopia has significantly improved access to the ART program, achieving and maintaining a satisfactory retention rate in long term remain a key challenge. As documented in some studies, carried out in Ethiopia as well as in other sub-Saharan countries, retention rates decrease over time. In the aforementioned article, conducted on 55 HFs in Ethiopia, Assefa et al showed that after 6, 12, and 24 months on ART retention rate was 80%, 74% and 68% respectively. Similar results were published in other studies, like a systematic review in sub-Saharan Africa by Fox and Rosen [10], showing retention in care declining from 80.2% at 12 months to 76.8% at 24 months and 72.3% at 36 months. Moreover, despite the involvement of the local CBOs in tracing the patients who missed their scheduled follow-up visit, the information on how many patients were traced and the reasons for not returning to visit were not strictly reported. For this reason patients lost of follow-up may also have died or have stopped taking ART.

The competitive advantage of the present study is that, differently from published reports where data collection has been prevalently retrospective, our retention rate calculations are based on a longitudinal observational study of a prospective cohort which is ongoing and will be followed for at least 5 years.

This article only provides preliminary results on a short period of time and with few participating HFs. Additional analyses will be implemented in the next future.

Supporting Information

S1 Data. Dataset for Table 1.
(SAV)

S2 Data. Dataset for Table 2.
(SAV)

S3 Data. Dataset for Table 3 and Fig 1.
(SAV)

Acknowledgments

We thank all the patients participating in this study, the health facilities and the CASA-project team.

CASA-Project Team

Raffaella Bucciardini, Istituto Superiore di Sanità, Rome, Italy; Hagos Godefay, Tigray Regional Health Bureau, Mekelle, Ethiopia; Stefano Vella, Istituto Superiore di Sanità, Rome, Italy; Loko Abraham, College of Health Sciences, Mekelle University, Mekelle, Ethiopia; Teshome Abegaz, College of Health Sciences, Mekelle University, Mekelle, Ethiopia; Vincenzo Fragola, Istituto Superiore di Sanità, Rome, Italy; Stefano Lucattini, Istituto Superiore di Sanità, Rome, Italy; Paola De Castro, Istituto Superiore di Sanità, Rome, Italy; Atakilt Halifom, Tigray Regional Health Bureau, Mekelle, Ethiopia; Roberta Terlizzi, Istituto Superiore di Sanità, Rome, Italy; Andrea Binelli, Istituto Superiore di Sanità, Rome, Italy; Eskedar Tadesse, College of Health Sciences, Mekelle University, Mekelle, Ethiopia; Micheal Berhe, College of Health Sciences, Mekelle University, Mekelle, Ethiopia; Katherina Pugliese, Istituto Superiore di Sanità, Rome, Italy; Luca Fucili, Istituto Superiore di Sanità, Rome, Italy; Massimiliano Di Gregorio, Istituto Superiore di Sanità, Rome, Italy; Marco Mirra,Istituto Superiore di Sanità, Rome, Italy; Teame Zegeye, Tigray Regional Health Bureau, Mekelle, Ethiopia; Tsigemariam Teklu, Tigray Regional Health Bureau, Mekelle, Ethiopia; Erika Olivieri, Istituto Superiore di Sanità, Rome, Italy. *Contact person*: *Raffaella Bucciardini*, raffaella.bucciardini@iss.it.

CASA-project Health Facilities

Ayder Hospital, Mekelle, Ethiopia: Hagos Asfaw, Hana Bekelle, Ataklti Birhane, Hadas Birhanu, Akberet Mengesha, Weldebirhan Teklu.

Alamata Health Center, Alamata, Ethiopia: Negasi Abay, Alemash Abebe, Molla Abreha, Yeshiwork Birhane, Eyob Hailu, Tshigie Kassa, Zinabu Kassa, Sindayo Tefera.

Mekelle Health Center, Mekelle, Ethiopia: Milka Asfaw, Slas Beyene, Kiros Haile, Kelel Tsegay.

Mehoni Health Center, Mehoni, Ethiopia: Mesele Adhena, Amina Beshir, Marlin Fisha, Abreha Goitom, Asefu Hindeya, Ashenafi Moges, Hafitu Siyoum, Gerezgiher Tsadik.

Author Contributions

Conceived and designed the experiments: RB SV HG LA A. Haile. Performed the experiments: RB VF TA SL ET MB KP AB LF MD. Analyzed the data: RB VF. Wrote the paper: RB SV HG PD VF. Designed the sofware used in analysis: TA SL LF MD MM. Administrative support: RT EO TT TZ. Local supervisor: A. Halifom.

References

1. Federal Democratic Republic of Ethiopia—Country Progress Report on HIV/AIDS Response, 2014 Available: http://www.unaids.org/sites/default/files/country/documents/ETH_narrative_report_2014.pdf. Accessed 22 July 2015.
2. Central Statistical Agency (Ethiopia) and ICF International: Ethiopia Demographic and Health Survey, Calverton, Maryland, USA: Central Statistical Agency and ICF International, 2011. Available: http://dhsprogram.com/pubs/pdf/FR255/FR255.pdf.
3. WHO, UNAIDS, UNICEF: Towards universal access: scaling up priority HIV/AIDS interventions in the health sector. Progress report 2011.Available: http://www.who.int/hiv/pub/progress_report2011/en/.
4. Ministry of Health (MOH): Accelerated access to HIV/AIDS treatment in Ethiopia: Road map 2004–2006. Addis Ababa, Ethiopia: Ministry of Health; 2004. Available: http://www.jourlib.org/references/57956.
5. Assefa Y, Kloos H. The public health approach to ART service scale-up in Ethiopia: the first two years of free ART, 2005–2007. Ethiop Med J 2008; 46(4):401–406. PMID: 19271406.
6. Federal HIV/AIDS Prevention and Control Office: Ethiopian multi- sectorial HIV/AIDS response performance report. Addis Ababa, Ethiopia; 2009.
7. World Health Organization (WHO). Consolidated guidelines on the use of antiretroviral drugs for treating and preventing HIV infection: recommendations for a public health approach. Geneva: World Health Organization;2013. Available: http://apps.who.int/iris/bitstream/10665/85321/1/9789241505727_eng.pdf.
8. World Health Organization (WHO). Retention in HIV programmes: Defining the challenges and identifying solutions. Geneva: World Health Organization;2012. Available: http://www.who.int/hiv/pub/meetingreports/retention_programmes/en/.
9. Assefa Y, Jerene D, Lulseged S, Ooms G, Van Damme W. Rapid Scale-up of Antiretroviral Treatment in Ethiopia: Successes and System-wide Effects. PLoS Med 2009; 6(4):e1000056. doi: 10.1371/journal.1000056 PMID: 19399154
10. Fox MP, Rosen S. Patient retention in antiretroviral therapy programs up to three years on treatment in Sub-Saharan Africa, 2007–2009: systematic review. Trop Med Int Health 2010;Suppl 1: :1–15. doi: 10.1111/j.1365–3156.2010.02508.xreview. PMID: 20586956.
11. Tassie JM, Baijal P, Vitoria MA, Alisalad A, Crowley SP, Souteyrand Y. Trends in retention on antiretroviral therapy in National programmes in low- income and middle-income countries. J Acq Immun Def Synd 2010; 54:437–441.doi: 10.1097/QAI.0b013e3181d73e1b PMID: 20351559.
12. Geng EH, Nash D, Kambugu A, Zhang Y, Braitstein P, Christopoulos KA, et al. Retention in care among HIV-infected patients in resource-limited settings: emerging insights and new directions. NIH Public Access. Curr HIV/AIDS Rep. 2010; 7(4):234–244. doi: 10.1007/s11904–010–0061–5 Review. PMID: 20820972.
13. World Health Organization (WHO). Country Statistics and Global Health Estimates by WHO and UN Partners. Ethiopia: WHO Statistical Profile. Available: http://www.who.int/gho/countries/eth.pdf?ua=1.
14. Melaku Z, Lamb MR, Wang C, Lulseged S, Gadisa T, Ahmed S et al. Characteristics and outcomes of adult Ethiopian patients enrolled in HIV care and treatment: a multi-clinic observational study" in contextualizing our findings. BMC Public Health, 2015; 15:462.
15. Assefa Y, Kiflie A, Tesfaye D, Mariam DH, Kloos H, Edwin W, et al.Outcomes of antiretroviral treatment program in Ethiopia: Retention of patients in care is a major challenge and varies across health facilities. BMC Health Serv Res. 2011; 11:81. doi: 10.1186/1472–6963–11–81 PMID: 21501509.
16. Wubshet M, Berhane Y, Worku A, Kebede Y, Diro E. High loss to follow-up and early mortality create substantial reduction in patient retention at antiretroviral treatment program in north-west Ethiopia.—International Scholarship Research Network-ISRN AIDS 2012; 14;2012:721720. E Collection 2012.
17. Federal Ministry of Health of Ethiopia: Guidelines for implementation of HIV/AIDS case management in Ethiopia. Addis Ababa, Ethiopia: FMOH; 2010. Available: http://www.etharc.org/resources/download/finish/33/83.
18. Assefa Y, Alebachew A, Lera M, Lynen L, Wouters E, Van Damme W. Scaling up antiretroviral treatment and improving patient retention in care: lessons from Ethiopia, 2005–2013. Global Health 2014; May 27; 10:43. doi: 10.1186/1744–8603–10–43PMID: 24886686.
19. Federal HIV/AIDS Prevention and Control Office: Monitoring and Evaluation Report, 2011/2012. Addis Ababa, Ethiopia: FHAPCO; 2013. Available: http://www.hapco.gov.et/.
20. Assefa Y, Kiflie A, Tekle B, Mariam DH, Laga M, Van Damme W. Effectiveness and acceptability of delivery of antiretroviral treatment in health centres by health officers and nurses in Ethiopia. J Health Serv Res Policy 2012; 17(1):24–29.doi: 10.1258/jhsrp.2011.010135PMID: 22096081.

21. Assefa Y, Lynen L, Wouters E, Rasschaert F, Peeters K, Van Damme W. How to improve patient retention in an antiretroviral treatment program in Ethiopia: a mixed-methods study. BMC Health Serv Res 2014; 29;14:45.
22. WHO: Task shifting: Rational redistribution of tasks among health workforce teams. Global recommendations and Guidelines. Geneva, Switzerland: WHO; 2007. Available: http://www.who.int/healthsystems/TTR-TaskShifting.pdf.
23. Callaghan M, Ford N, Schneider H. Systematic review of task-shifting for HIV treatment and care in Africa. Hum Resour Health. 2010; 31; 8:8.
24. Etienne M, Burrows L, Osotimehin B, Macharia T, Hossain B, Redfield RR, et al. Situational analysis of varying models of adherence support and loss to follow up rates; findings from 27 treatment facilities in eight resource limited countries. Trop Med Int Health 2010; 15:76–81.15; doi: 10.1111/j.1365–3156.2010.02513.xPMID: 20586964.
25. Wouters E, Van Damme W, van Rensburg D, Masquillier C, Meulemans H. Impact of community-based support services on antiretroviral treatment programme delivery and outcomes in resource-limited countries: a synthetic review. BMC Health Serv Res 2012; 12:194. doi: 10.1186/1472–6963–12–194PMID: 22776682.
26. Massaquoi M, Zachariah R, Manzi M, Pasulani O, Misindi D, Mwagomba B, et al.Patient retention and attrition on antiretroviral treatment at district level in rural Malawi. Trans R Soc Trop Med Hyg 2009; 103(6):594–600.
27. Rich ML, Miller AC, Niyigena P, Franke MF, Niyonzima JB, Socci A, et al. Excellent clinical outcomes and high retention in care among adults in a commuty-based HIV treatment program in Rural Rwanda. J Acquir Immune Defic Syndr 2012; 59: e35–e42 doi: 10.1097/QAI.0b013e31824476c4 PMID: 22156912.
28. Mwai GW, Mburu G, Torpey K, Frost P, Ford N, Seeley J. Role and outcomes of community health workers in HIV care in sub-Saharan Africa: a systematic review. J Int AIDS Soc. 2013; Sep 10; 16:18586. Review.
29. Schneider H, Lehmann U. Lay health workers and HIV programmes: implications for health systems. AIDS Care 2010; 22 Suppl 1:60–7. doi: 10.1080/09540120903483042PMID: 20680861.
30. Wright J, Walley J, Philip A, Pushpananthan S, Dlamini E, Newell J, et al. Direct observation of treatment for tuberculosis: a randomized controlled trial of community health workers versus family members. Trop Med Int Health. 2004; 9(5):559–565.PMID: 15117299.
31. Arem H, Nakyanjo N, Kagaayi J, Mulamba J, Nakigozi G, Serwadda D, et al. Peer health workers and AIDS care in Rakai, Uganda: a mixed methods operations research evaluation of a cluster-randomized trial. AIDS Patient Care STDS 2011; 25(12):719–724. doi: 10.1089/apc.2010.0349 PMID: 21391828.
32. Selke HM, Kimaiyo S,Sidle JE, Vedanthan R, Tierney WM, Shen C, et al. Task-shifting of antiretroviral delivery from health care workers to persons living with HIV/AIDS: clinical outcomes of a community-based program in Kenya. J Acquir Immune Defic Syndr. 2010; 55(4):483–490.doi: 10.1097/QAI.0b013e3181eb5edbPMID: 20683336.
33. Hart JE, Jeon CY, Ivers LC, Behforouz HL, Caldas A, Drobac PC, et al. Effect of directly observed therapy for highly active antiretroviral therapy on virologic, immunologic, and adherence outcomes: a meta-analysis and systematic review. J Acquir Immune Defic Syndr. 2010; 54:167–179. doi: 10.1097/QAI.0b013e3181d9a330 Review. PMID: 20375848.
34. Barnighausen T, Chaiyachati K,Chimbindi N, Peoples A, Haberer J, Newell ML, et al. Interventions to increase antiretroviral adherence in sub-Saharan Africa: a systematic review of evaluation studies. Lancet Infect Dis. 2011; 11(12):942–51. doi: 10.1016/S1473–3099(11)70181–5 Review. PMID: 22030332.
35. Fatti G, Meintjes G, Shea J, Eley B, Grimwood A. Improved survival and antiretroviral treatment outcomes in adult receiving community-based adherence support:5-year results from a multicentre cohort study in South Africa. J Acquir Immune Defic Syndr 2012; 61(4):e50–e58. doi: 10.1097/QAI.0b013e31826a6aeePMID: 22842842.
36. Farmer P, Leandre F, Mukherjee JS, Claude M, Nevil P, Smith-Fawzi MC et al. Community-based approaches to HIV treatment in resource-poor settings. Lancet. 2001; 358:404–409.PMID: 11502340
37. Center for Disease Control and Prevention (CDC) 2013. Differences Between HIV-Infected Men and Women in Antiretroviral Therapy Outcomes—Six African Countries, 2004–2012. Morbidity and Mortality Weekly Report, November 29, 2013 / 62(47);946–952. Available: http://www.cdc.gov/mmwr/preview/mmwrhtml/mm6247a2.htm.
38. Muula AS, Ngulube TJ, Siziya S, Makupe CM, Umar E, Prozesky HW,et al. Gender distribution of adult patients on highly active antiretroviral therapy (HAART) in Southern Africa: a systematic review. BMC Public Health 2007; 7:63 doi: 10.1186/1471–2458–7–63.17459154 PMID: 17459154

39. Taylor-Smith K, Tweya H, Harries A, Schoutene E, Jahn A. Gender differences in retention and survival on antiretroviral therapy of HIV-1 infected adults in Malawi. Malawi Medical Journal 2010; 22(2): 49–56.PMID: 21614882.

40. Cornell M, Myer L, Kaplan R, Bekker LG, Wood R. The impact of gender and income on survival and retention in a south African antiretroviral therapy programme. Trop Med Int Health, 2009; 14(7): 722–731.

41. World Health Organization (WHO) Global tuberculosis report 2012. Available: http://apps.who.int/iris/bitstream/10665/75938/1/9789241564502_eng.pdf.

42. UNAIDS 2010;Getting to zero: 2011–2015 Strategy.Geneva, Joint United Nations Programme on HIV/AIDS(UNAIDS) 2010. Available: http://www.unaids.org/sites/default/files/sub_landing/files/JC2034_UNAIDS_Strategy_en.pdf.
